# Role of Cytokinins in Senescence, Antioxidant Defence and Photosynthesis

**DOI:** 10.3390/ijms19124045

**Published:** 2018-12-14

**Authors:** Martin Hönig, Lucie Plíhalová, Alexandra Husičková, Jaroslav Nisler, Karel Doležal

**Affiliations:** 1 Department of Chemical Biology and Genetics, Centre of the Region Haná for Biotechnological and Agricultural Research, Faculty of Science, Palacký University, Šlechtitelů 27, CZ-783 71 Olomouc, Czech Republic; martin.honig@upol.cz (M.H.); jaroslav.nisler@gmail.com (J.N.); karel.dolezal@upol.cz (K.D.); 2 Laboratory of Growth Regulators, Centre of the Region Haná for Biotechnological and Agricultural Research, Palacký University & Institute of Experimental Botany ASCR, Šlechtitelů 27, CZ-783 71 Olomouc, Czech Republic; 3 Department of Biophysics, Centre of the Region Haná for Biotechnological and Agricultural Research, Faculty of Science, Palacký University, Šlechtitelů 27, CZ-783 71 Olomouc, Czech Republic; alexandra.husickova@upol.cz

**Keywords:** cytokinin, derivative, antisenescent, antioxidant, structure and activity relationship, genes, antioxidant enzymes, photosynthesis, plant defence

## Abstract

Cytokinins modulate a number of important developmental processes, including the last phase of leaf development, known as senescence, which is associated with chlorophyll breakdown, photosynthetic apparatus disintegration and oxidative damage. There is ample evidence that cytokinins can slow down all these senescence-accompanying changes. Here, we review relationships between the various mechanisms of action of these regulatory molecules. We highlight their connection to photosynthesis, the pivotal process that generates assimilates, however may also lead to oxidative damage. Thus, we also focus on cytokinin induction of protective responses against oxidative damage. Activation of antioxidative enzymes in senescing tissues is described as well as changes in the levels of naturally occurring antioxidative compounds, such as phenolic acids and flavonoids, in plant explants. The main goal of this review is to show how the biological activities of cytokinins may be related to their chemical structure. New links between molecular aspects of natural cytokinins and their synthetic derivatives with antisenescent properties are described. Structural motifs in cytokinin molecules that may explain why these molecules play such a significant regulatory role are outlined.

## 1. Introduction

Naturally occurring cytokinins (CKs) are purine based plant growth regulators that influence almost all of the developmental stages of plant life, e.g., development of vasculature, differentiation of embryonic cells, maintenance of meristematic cells, shoot formation and leaf senescence delay. CKs were first discovered as substances that promoted cell division in tissue cultures in the presence of auxin [1]. Naturally occurring CKs are purine based molecules that are substituted at the C6 atom either by an isoprene side chain (ISCKs) or aromatic core (ARCKs). ISCKs are represented by naturally occurring 6-(*E*)-4-hydroxy-3-methylbut-2-enylaminopurine (*trans*-zeatin, *t*Z), 6-(*Z*)-4-hydroxy-3-methylbut-2-enylaminopurine (*cis*-zeatin, *c*Z) and 6-(2-isopentenylamino)purine (iP). ARCKs include 6-benzylaminopurine (BAP), 6-furfurylaminopurine (kinetin, Kin) and *o-*, *m-* and *p-*hydroxylated or methoxylated derivatives of BAP, called topolins [2]. Although BAP and Kin are not traditionally considered as naturally occurring CKs, exogenous treatment with them showed such strong effects on plant tissues that these molecules were often used experimentally and formed the basis of the first generation of synthetic ARCKs [3]. Nucleosides, nucleotides and other sugar conjugates of many CKs have been found in plants, and metabolic networks exist for their interconversions [1]. Moreover, the presence of a purine moiety enables a number of possible modifications, including substitution at the C2, N1, N3, C6, N7 or N9 atoms of the original purine heterocycle [3]. The family of CK compounds includes a large array of natural and synthetic purine and phenylurea derivatives. The most effective phenylurea CK is thidiazuron (TDZ) [4]. The majority of these compounds are not considered as naturally occurring, however they possess significant CK activity [1]. The action of CKs is often influenced by interaction with other hormones, e.g., auxins or ethylene [4]. One of the most valued features of CK action and the focus of this review is regulation of senescence, especially senescence delay, owing to the potential for economic benefits [5,6,7,8]. Leaf senescence is the final step in leaf development and is often accompanied by colour changes from green to yellow or brown [9]. Leaf yellowing is not only age dependent, it can also be induced by a number of other factors, including biotic stress, mechanical damage, harvesting and environmental stress [10]. Leaf senescence seems to be directly related to a decrease in CK concentration and activity. Generally, CKs are more effective when applied to detached plant organs [5,11], however they can also delay the senescence of attached leaves. CKs are also effective in delaying the breakdown of chlorophyll, suggesting that they may play a role in maintaining the photosynthetic apparatus of plants [5]. Many of the biological activities of CKs in plants can be explained by their involvement in cellular oxidative stress, and the antioxidant capacity of these molecules has already been described [12]. Leaf senescence is accompanied by a gradual decline in antioxidants and an increase in reactive oxygen species (ROS) and content of malondialdehyde (a decomposition product of lipid peroxidation) in certain plants [13,14]. Increased lipid peroxidation and H_2_O_2_ formation have been demonstrated along with a decline in the activities of enzymes, such as catalase (CAT) and ascorbate peroxidase (APX), as well as glutathione (GSH) content during senescence in pea and *Arabidopsis* leaves [13,15].

Regulation of the onset of senescence is important owing to its impact on dynamic nutrient relocations during the degradation of cellular components [9]. Leaf senescence can be regulated in a number of ways that include ethylene production and expression of senescence associated genes (SAGs). Important roles are played by antioxidant enzymes, such as CAT, APX and superoxide dismutase (SOD), and compounds related to oxidative stress, such as phenolic acids, flavonoids and H_2_O_2_. Changes in chlorophyll are inseparably linked to photosynthesis. Exogenous treatment with CKs and their derivatives or genetic engineering to create plants that overproduce CKs can lead to significant senescence delay. In this review, we focus on the main mechanisms of action by which CKs and their derivatives delay leaf senescence.

## 2. Antisenescent and Antioxidant Activity of Natural Cytokinins (CKs), Kinetin (Kin) and 6-Benzylaminopurine (BAP)

The first evidence for the antisenescent activity of CKs was provided by Richmond and Lang [16] in an experiment testing exogenous application of Kin on excised leaves of cocklebur plants (*Xanthium pensylvanicum*) and tobacco. The results showed that this led to re-greening in yellowing tobacco leaves, however not in *Xantium pensylvanicum* [17]. The need to measure the ability of CKs in retarding chlorophyll degradation in various plant tissues prompted the development of several different senescence bioassays. CKs can be applied as droplets on leaves or by floating leaves (or their parts) on a CK solution or by inserting the base of a detached leaf into a CK solution [18]. The latter method has been optimized with wheat leaf segments [19], known as the wheat leaf senescence assay (WLSA), and is widely used to assess the antisenescence properties of CKs. Briefly, a tip section of a seven-day-old wheat leaf is inserted into a solution of CK and is placed in the dark for five days. Afterwards, any chlorophyll remaining in the leaf tips is extracted by 80% ethanol and its content is determined spectrophotometrically at 665 nm [19].

**Figure 1:**
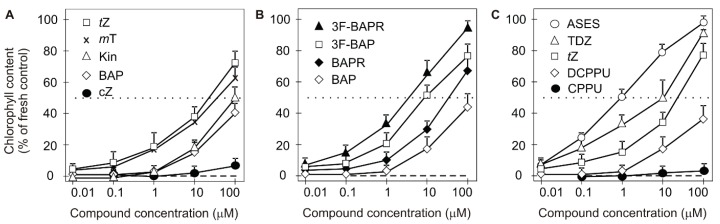
Evaluation of the biological activity of (**A**) classical cytokinins, (**B**) 3-fluoro derivatives of BAP and BAPR and (**C**) urea based cytokinins in the wheat leaf senescence assay performed in the dark (according to [19]). The dotted line indicates where the chlorophyll content in the leaves is 50% of that in fresh control leaves, while 100% represents the chlorophyll content in fresh control leaves. Dashed lines indicate values for the control treatment (DMSO control) with no added compound. Error bars show the S.D. of the mean for four replicate determinations.

Comparison of the antisenescent activity of different CK free bases in the WLSA performed according to protocol described by Holub et al. showed that *t*Z clearly had the highest activity, followed by 6-(3-hydroxybenzylamino)purine *(meta-t*opolin, *m*T), of which its activity was only marginally lower than that of *t*Z [19]. BAP and Kin had similar activity in the WLSA, however both compounds were less active than *t*Z and *m*T [19]. In contrast, 6-(2-hydroxybenzylamino)purine *(ortho-*topolin, *o*T), *cis*-zeatin and iP were considered as CKs with low or no antisenescent effect [19,20,21]. The activities of *t*Z, *c*Z, *m*T, BAP and Kin in the WLSA are compared in Figure 1A. Holub et al. [19] further showed that 9-ribosides of *t*Z, BAP, *m*T and *o*T were more active than their free bases [19]. In the case of *t*Z and *trans*-zeatin riboside (*t*ZR), this was in accord with results of Spíchal et al. [22] and Kim et al. [23]. Both *t*Z and *t*ZR strongly activated the *Arabidopsis* CK receptor AHK3, which was shown by Kim et al. [23], to play a crucial role in CK-mediated leaf longevity through phosphorylation of the CK response regulator ARR2. Conversely, benzylaminopurine riboside (BAPR) and *m*TR did not activate the AHK3 receptor [22], however they were found to be active in the WLSA [19]. Doležal et al. [24] suggested that ARCKs, especially *o-*, *m-*methoxy and halogeno derivatives of BAP, may protect against the degradation of the photosynthetic apparatus. This was based on findings that substituted ARCKs had antisenescent activities similar to or higher than those of *t*Z yet activated *Arabidopsis* CK receptors AHK3 and CRE1/AHK4 only weakly [22,24].

Better understanding of CK involvement in plant senescence was provided by analyses of CK concentration levels through plant development and tissues. These studies revealed an inverse relationship between CK levels and the progression of senescence in a variety of tissue and plant species [5,25]. The importance of naturally occurring ISCK sugar conjugates, dihydrozeatin riboside (DHZR) and zeatin riboside (ZR), in xylem sap during plant senescence was identified in experiments on soybean [26]. CK levels in the xylem sap of soybean (*Glycine max*) decreased rapidly with the onset of leaf senescence [26]. Reduction in endogenous CKs (zeatin (Z), dihydrozeatin (DHZ), ZR, DHZR) was also observed in tobacco leaves during the progression of leaf senescence [27]. However, in exogenous CK application experiments, it was unclear how much of the hormone was actually transported to and taken up by the plant tissues. Moreover, these factors may differ in different species or under different experimental conditions [25]. Podlešáková et al. published a study on the acropetal transport of 6-(3-methoxybenzylamino)purine (*meta*-methoxytopolin, *m*MT), 9-(4-chlorobutyl) and 9-(tetrahydropyran-2-yl, THP) derivatives of *m*MT. It was shown that the derivatives allowed the gradual release of the active base and had a significant impact on the distribution and amount of endogenous ISCK in various plant tissues [28]. Although a relationship between CK levels and senescence progression was found, the regulatory role of CKs in plant senescence was clarified using transgenic plants. Cloning of the *Agrobacterium* IPT (isopentenyl transferase) gene encoding a CK producing enzyme provided a way to genetically engineer plants in which endogenous levels of these hormones could be increased [6]. The effect of CK treatment on these genetically altered plants is discussed in the text below.

Since naturally occurring CKs contain heterocycles in their structure and are able to regulate leaf senescence, questions were raised about their possible antioxidant activity. Increased levels of H_2_O_2_, lipid peroxidation and membrane leakiness were observed during leaf senescence in tobacco, rice, pea, sunflower and barley [13,29,30,31,32,33]. External application of BAP increased the activity of CAT and APX enzymes and reduced the level of H_2_O_2_ during dark senescence of wheat leaves. It was suggested that externally applied CKs may protect the cell membranes and photosynthesis system from oxidative damage during the delay of senescence in the dark [34,35]. However, in dexamethasone-inducible IPT transgenic tobacco plants with increased levels of endogenous CKs, higher concentrations of H_2_O_2_ were detected, especially in chloroplasts. Moreover, the increased levels of H_2_O_2_ resulted into elevated lipid peroxidation [36]. It was shown by the analysis of the end-product of lipid peroxidation, malondialdehyde (MDA), that lipid peroxidation is not associated with leaf senescence in stay-green tobacco plants [14]. 

## 3. Antisenescent and Antioxidant Activity of C2, C8 and N9 Purine-Based CK Derivatives

Since ISCKs and ARCKs appear to be crucial to senescence regulation, a number of their derivatives have been prepared to study the structure and activity relationship of these processes. In 1989, Zhang et al. measured the antisenescent activity of nine substituted CK derivatives using soybean leaf discs. Of the 14 BAP derivatives tested, including 9-alanine, 9-tetrahydropyranyl and 9-tetrahydrofuranyl, the last two derivatives were found to be the most active in the bioassay due to their great stability and ability to gradually release free BAP [37]. In the same year, another series of 9-substituted derivatives of four common naturally occurring CKs were tested in the soybean callus assay. Specifically, 9-(2-Carboxyethyl), 9-(2-carbo-*t*-butoxyethyl) and 9-(2-nitroethyl) derivatives of *t*Z, *c*Z, DHZ and iP all reduced the biological activity of the parent compounds [38]. Another 33 6-BAP-9-tetrahydropyranyl (THP) and 9-tetrahydrofuranyl (THF) derivatives with variously positioned hydroxyl- and methoxy-functional groups on the benzyl ring were prepared by Szüčová et al. [39]. The majority of the prepared derivatives showed higher antisenescent activity than the standard BAP or parent CKs (exceptions were derivatives substituted in *p*- positions or derivatives with a blocked N6-H atom) [39,40,41]. In contrast, derivatives shorter by a CH_2_ group, i.e., 6-anilinoaminopurine derivatives, prepared by Zatloukal et al. were shown to be inactive in the WLSA [42]. Eight Kin derivatives were prepared by the addition of various halogenoalkyls, ethoxyethyl, carboxyl or THF groups to the N9 atom of the purine moiety. Some of these derivatives, especially those bearing THF, ethoxyethyl and chloroethyl, were highly active in the WLSA (reaching 110–131% of the original Kin) [34]. Moreover, compounds that were active in the senescence bioassay significantly reduced peroxidation of membrane lipids under dark conditions [34].

In addition to N9-substitution, a number of BAP derivatives were prepared by Doležal et al. with substitutions (halogen(s), methyl or methoxy group(s)) in various positions on the N6-benzyl ring [24]. Among them, 3-fluoro-BAP (3F-BAP) exhibited the highest antisenescent activity in the WLSA (Figure 1B). Some derivatives also possessed increased antisenescent activity compared to BAP (for details, see [24]). One year later, Doležal et al. published similar work on the preparation and testing of BAPR derivatives [43]. Unsurprisingly, BAPRs were found to be more effective than the corresponding BAPs (Figure 1B). Specifically, 3F-BAPR was the most active compound in the WLSA, with antisenescent activity up to 220% of that of BAP [43] (Figure 1B). Vylíčilová et al. [44] prepared 14 halogenated derivatives of BAPR by the addition of chlorine at the C2 atom of purine. The compounds exhibited increased antisenescence activity in the WLSA compared to BAPs, however lower activity than BAPR derivatives. In other words, chlorine substitution at C2 decreased the antisenescent activity of BAPR and counteracted the positive activity of ribose. The antisenescent activity of 2-chloro-6-(3-fluorobenzylamino)purine-9-riboside (2cl-3F-BAPR) is thus similar to the activity of 3F-BAP. For clarity, we have again compared the antisenescence activity of BAP, BAPR, 3F-BAP a and 3F-BAPR in one WLSA according to Holub et al. [19] (Figure 1B). In addition, Vylíčilová et al. further showed that the introduction of chlorine to the C2 position of the purine moiety dramatically decreased the toxicity of several toxic previously prepared BAPs and BAPRs [24,43,44]. The antisenescent activity of 2-chloro-6-(3-fluororbenzylamino)purine-9-riboside (2Cl-3F-BAPR) was similar to the activity of 3F-BAP. However, this study of Vylíčilová et al. was the first to uncover possible targets of the BAPR derivatives in the inhibition of chlorophyll degradation [44]. Genome-wide expression profiling showed that the synthetic halogenated derivatives of BAPR affected transcription of a unique combination of genes coding for components of the photosystem II (PSII) reaction centre, light-harvesting complex II (LHCII) and oxygen-evolving complex, as well as several stress factors responsible for regulating photosynthesis and chlorophyll degradation [44].

More recently, 11 Kin derivatives were prepared and tested in the WLSA. The presence of an oxygen atom in the furan ring was shown to be a critical structural motif for slowing the breakdown of chlorophyll in the WLSA. Replacement of the oxygen atom by sulphur or carbon resulted in decreased antisenescent activity. On the other hand, saturation of the furan ring did not have such a negative impact on the antisenescent activity. Moreover, 9-THF substitution had no effect or slightly improved the antisenescent activity of 6-(tetrahydrofuran-2-ylmethyl)aminopurine, Kin and 6-(thiophen-2-ylmethyl)aminopurine (thiokinetin). The addition of chlorine at the C2 atom of purine moiety lowered or completely reduced the antisenescent activity of the prepared compounds. Furthermore, the prolongation of the C-bridge carrying the N6-substituent of 6-(2-thiophen-2-ylethyl)aminopurine led to the complete loss of the antisenescent activity in the WLSA [45].

Synthetic CK analogues, particularly 6-alkynyl and 6-alkenylaminopurines, some of which were also substituted at the N9 atom of the purine moiety, have been tested for their antioxidant activity as potential diphenylpicrylhydrazyl (DPPH) scavengers and as inhibitors of 15-lipoxygenase enzyme, together with naturally occurring CKs BAP, Kin and *t*Z [46]. Whereas naturally occurring CKs were unable to scavenge DPPH, some of the prepared compounds were significantly more active than BAP, Kin and *t*Z. The most active compounds were 6-(3-thienylethenyl)purine, with 18% scavenging activity after 15 min, however also derivatives incorporating 2-furyl in their structure [46]. 

Substitution of the C6 atom of the purine moiety seems to be crucial for the antisenescent properties of the prepared derivatives. A unique example of an inactive CK is isopentenylaminopurine (iP). Neither iP nor its derivatives are active in the WLSA. To test this, a series of N6-(3-methylbut-2-en-1yl)amino)purine (iP) derivatives substituted at the N9 atom of the purine moiety was prepared in 2011 [21]. As expected, none of these compounds were active in the WLSA, although the prepared compounds were evaluated as active CKs in other CK bioassays [21].

Recently, another 58 CK derivatives substituted at the C8 atom of the purine moiety, 27 of which were 9-tetrahydropyranyl precursors of CKs, were published by Zahajská et al. [47]. The introduction of C8 substitution led to a decrease or even the complete loss of antisenescent activity in the majority of compounds compared to the corresponding free bases. However, 6-benzylamino-7,9-dihydro-8H-purin-8-one (8-oxo-BAP) exhibited higher activity than BAP by 34%. Concurrently, among Kin derivatives, methoxy- and 2-hydroxyethyloxy-C8 substituents did not decrease the activity of Kin. With some exceptions, compounds with a 9-(tetrahydropyran-2-yl) protective group exhibited even lower activity in the WLSA than their THF deprotected analogues [47]. This observation is in contrast to previous studies, in which the introduction of 9-THP or 9-THF protective groups was found to not decrease the antisenescent activity of the corresponding free bases [37,40,45,48].

According to the above, the crux of the antisenescent activity of CKs appears to be appropriate substitution at the N6 atom. Concurrently, the N6-H hydrogen must stay unsubstituted. Substituents at nearby atoms, such as N6, must contain an oxygen atom incorporated into oxo-, hydroxyl or methoxy groups and/or halogen atoms, such as fluorine or chlorine. Further, *t*Z, Kin and topolins are typical examples of CKs shown to be very active in antisenescent bioassays. Substitution at C8 drastically reduces the antisenescent properties of nearly all, even the active CKs, with one exception: compounds substituted by oxygen at C8 remain active. Substituents at the N9 atom can vary, however they do not normally affect the antisenescent properties much if the N6 substitution is “antisenescent”, with the exception of ribosides, which in some cases are more active. Questions arise concerning whether other sugars at the N9-position may have a similar or even better effect. The structural trends described above show that the antisenescent effects are probably connected with the presence of electronegative atoms, oxygen and halogens, which are close to the N6 and/or N9 atoms of purine. Both the N6H and N7H hydrogen atoms are present and “free to operate” in all active compounds. The second condition is the presence of –O–, –OH, –X or –OCH_3_ substitutions close to this area. These two factors can lead to the concentration of electron density and increase of antisenescent activity in such compounds. The electron density enhancement at these strategic atoms is most probably responsible for the antisenescent effects.

Based on the available literature and a large amount of data, it is evident that the antisenescent properties of some CK derivatives are not directly associated with their CK activity. Moreover, iP and its derivatives are active CKs, however they are inactive in the WLSA [21,47]. Conversely, some derivatives of BAP, BAPR, 9-THF (THP)—BAP and Kin exhibit low CK activity at the receptor level, lower or average activity in other CK bioassays, however increased antisenescence activity [34,39,43]. 

An example of such phenomena—the presence of an oxygen containing group and lack of relation of CK activity to the antisenescent activity—was provided recently by Nisler et al. [49]. They showed that urea derived compounds are extremely active in the WLSA (Figure 1C) if they contain a methoxy group (a compound called ASES), hydroxyl or other electron-rich groups. Interestingly, the compounds also needed to have a second NH not substituted (like the N6H in purines) to be active in the WLSA. It was also shown that the compounds exhibited none or very low CK activity in almost all CK assays. Analysis of chloroplast membrane proteins showed that one way in which these urea derivatives delay senescence is by inhibition of photosystem II degradation [49]. This study agrees with that of Vylíčilová [44] and, at the protein level, supports and complements results at the transcriptional level.

Finally, it is worth noting that according to the literature, CKs (as well as other compounds with antisenescent properties) inhibit chlorophyll degradation by several mechanisms that may act synergistically. Some have been identified and well described, whereas others await discovery.

## 4. Antisenescent Activity of Urea Based CKs and Their Derivatives

Urea based compounds represent another class of highly active synthetic CKs. The best known are TDZ, *N*-(2-chloropyridin-4-yl)-*N*′-phenylurea (phenylurea, CPPU) and *N*-(2,6-dichloro-pyridin-4-yl)-*N*′-phenylurea (DCPPU). So far, TDZ appears to be the best representative of the synthetic CKs in terms of CK receptor activation [22], promotion of CK dependent callus growth [50,51] and, most importantly, inhibition of plant senescence [52,53,54,55]. In most CK bioassays, including the WLSA, TDZ exhibits even higher activity than *t*Z [22,49]). This may be due to a number of reasons, e.g., TDZ exhibits strong CK activities in several bioassays, however in contrast to *t*Z, cannot be degraded by cytokinin oxidase dehydrogenase (CKX, a key enzyme involved in CK degradation in plants [56,57]). TDZ cannot be deactivated by *O*-glucosylation and can maintain a higher content of endogenous CKs in plant tissue by inhibiting the function of CKX [58,59,60]. Owing to its high CK and particularly antisenescence activity, TDZ has been extensively studied and used to prolong the life of cut flowers [55]. In this field, no other substance has received as much attention as TDZ.

Several mechanisms for explaining how TDZ delays plant senescence have been shown in studies of various plant species [61]. In *Matthiola incana* cut flowers, TDZ reduced stress responses by inhibiting abscisic acid production, resulting in a higher content of chlorophyll and carotenoids [61] in stem leaves. Like ethylene, abscisic acid promotes senescence [62], occurs as a response to stress and is considered to be a marker of stress-induced senescence [63]. It was further shown that in *Pelargonium* cuttings, TDZ induced strong expression of PhETR1 (a negative acting ethylene receptor gene), thus decreasing the sensitivity of *Pelargonium* leaves to ethylene [64]. Ethylene is known to accelerate and accompany senescence [65,66]. In *Pelargonium zonale*, TDZ was shown to increase the levels of APX and SOD, which are enzymes of the antioxidant defence system [67]. Another complex study was carried out on stems and florets of cut *Chrysanthemum morifolium* plants. Both BAP and TDZ were found to increase the activity of antioxidant enzymes SOD and peroxidase (POD), reduce production of H_2_O_2_, minimize lipid peroxidation and maintain high levels of sugars in cut stems and florets. Treated plants also showed increased water uptake and prolonged the post-harvest quality of florets and leaves. In this study, TDZ again exhibited higher activity and in lower concentrations than BAP [68]. The antisenescent activity of CPPU, DCPPU, TDZ and ASES was compared in one WLSA (Figure 1C) according to Holub et al. [19].

To conclude, from the literature reviewed, it appears that TDZ delays senescence in plants by the same mechanisms as described previously for purine-derived CKs. However, its activity is higher, most probably because it cannot be enzymatically inactivated.

## 5. Ability of CKs and CK Derivatives to Improve the Antioxidant Capacity and Secondary Metabolite Content

CKs and their derivatives are often prepared for plant biotechnological applications and preferentially used in tissue cultures [3]. When particular CKs or their derivatives are employed in the micropropagation media of species with known antioxidant properties, plants exhibit increased antioxidant capacity through the explants grown [69]. In particular, a number of medicinal plants and often those high in antioxidants must be grown using micropropagation techniques due to their endangered status in nature. Such effects have been observed during the tissue culture and acclimatization of *Merwilla plumbea*, a plant widely used in traditional African medicine in Southern Africa and currently threatened in the wild because its medicinal use includes the bulbs [70]. Five CKs and their derivatives were evaluated during *M. plumbea* micropropagation: BAP, iP, *m*T, 3-*m*TR and 6-(3-methoxybenzyl)-9-(tetrahydropyran-2-ylamino)purine (*meta*-methoxytopolin THP, *m*MTHP) [70]. The antioxidant activity and phenolic acid content during the tissue culture and acclimatization of the plantlets were determined. The findings indicated that the phytochemical content during in vitro propagation of *M. plumbea* were influenced by the CKs and the majority of the phenolic acids were higher in the tissue culture than in the acclimatized plantlets [70]. Similar effects on the secondary metabolite content were found in a study on micropropagated “Williams” banana. However, the activity of specific compounds differed for different plant species [69]. The influence of CKs from tissue culture media on flavonoid levels of micropropagated plantlets is important as flavonoids have been identified as compounds that are able to scavenge free radicals. [71]. TDZ was found to significantly influence shoot multiplication and accumulation of secondary metabolites in *Scutellaria altissima* culture. *Scutellaria* plants express several pharmacologically and clinically important phytochemicals [72]. In particular, the flavonoid verbascoside possesses strong anti-inflammatory, antibacterial, antiviral and antioxidant activity [73]. Under the conditions used in the study, 2.5 times more flavonoids and six-times more verbascoside were accumulated in shoots that were grown on the medium supplemented with CKs than in shoots that were grown on CK free medium [74]. In further work on *Scutellaria altisima* explants, a higher level of other important flavonoids, such as baicalin and wogonoside, were observed in 12-week-old micropropagated plants. The shoot cultures were grown on MS agar medium that was supplemented with BAP [75]. Wogonoside in particular is interesting as this flavonoid is very active against lipid peroxidation [76]. The medicinally important plant *Eucomis autumnalis* has been treated with 0.01, 0.1 and 10 µM of 2-chloro-6-(3-methoxyphenylamino)purine (INCYDE) and CK antagonist 6-(2-hydroxy-3-methylbenzylamino)purine (PI-55) alone or in combination with BAP or naphthaleneacetic acid and the antioxidant response was evaluated in 10-week-old in vitro regenerates [77]. The levels of phytochemicals, especially those of flavonoids, were significantly affected by both tested compounds PI-55 as well as INCYDE. Besides, INCYDE application significantly increased the antioxidant activity of *E. autumnalis* in the DPPH test. On the other hand, the *beta*-carotene test was unaffected by INCYDE, however it was enhanced by BAP when it was used as a control [77]. Significantly improved antioxidant activity in the oxygen radical absorbance capacity (ORAC) assay was observed after one application of INCYDE in field-grown lettuce [78]. Both Kin and BAP enhanced the production of hypericins in *Hypericium maculatum* and hyperforin in *H. hirsutum* shoot cultures [79]. Naphthodianthrones (hypericin and pseudohypericin) and phloroglucinol hyperforin are valuable compounds that are associated with antiviral, antioxidant and other biological activities [80]. The increased flavonoid content and antioxidant activity of the common European herb sage (*Salvia officinalis* L.) has also been assessed during micropropagation [81]. The effects of four different CKs, i.e., TDZ, BAP, Z and iP, were evaluated. Levels of phytochemicals apigenin and its derivatives apigenin 7-methyl ether, scutellarein 6-methyl ether, scutellarein 6,7-dimethyl ether and luteolin were found to be comparable to those measured in plants grown on media without added CKs. On the other hand, BAP added to the media caused the production of hardened plants that successfully adapted in ex vitro conditions [81].

Aside from the above effects, CKs and their derivatives are able to influence the activity of antioxidant enzymes [35,82]. It was observed that the application of BAP prevents the degradation of chlorophyll in wheat senescent leaves and increases the activity of enzymes CAT and APX [35,83,84,85]. Increased SOD activity was observed after addition of *t*ZR to the grass *Agrostis palustris* [86]. BAP also increased levels of the APX enzyme after four and six days’ incubation of wheat leaves [35]. Application of BAP reduced H_2_O_2_ accumulation and lipid peroxidation of *Litchi* [83]. Furthermore, higher activity of SOD, CAT and APX and DPPH radical scavenging capacity were found in BAP treated *Litchi*. Data showing the influence of antioxidant compounds in different plant species are summarized in Table 1. It is apparent that the activity of individual CKs varies depending on the plant species and secondary metabolite group. However, whereas H_2_O_2_ and lipid peroxidation levels usually decrease after CK treatment, antioxidant enzymes CAT, APX and SOD, and antioxidant secondary metabolites, such as phenolic acids and flavonoids, increase.

## 6. CKs and (A)Biotic Stress Responses

CKs play an important and complex role in abiotic stress responses [93]. Endogenous CK levels decrease when the plant is under abiotic stress, such as mineral, salt or drought stress. Transgenic plants overexpressing an IPT gene under the control of a maturation and drought-induced promoter were shown to recover from drought [94]. In addition, CKs were shown to enhance immunity to biotic stress [94]. Some pathogens are able to “use” host plants to produce high levels of CKs, such as the gram-negative bacterium *Agrobacterium tumefaciens* [95]. *A. tumefaciens* uses AHK3 and AHK4-dependent transcription reprogramming to make host cells more receptive to infection [96,97]. A similar situation occurs during infection by the gram-positive bacterium *Rhodococcus fascians*, which uses a higher CK production pool for leaf gall formation and extending the ectopic growing shoot primordia [97,98,99]. Some fungal pathogens also produce CKs, such as the ergot fungus *Claviceps purpurea*, which infects the ovaries of rye [100]. These pathogens reprogramme the biosynthesis of CKs for more extensive infection. On the other hand, exogenous application of CKs affects salicylic acid mediated defence responses, e.g., in *Pseudomonas syringae* pv. tomato [97,101]. 

CKs have been suggested to be instrumental in mediating host susceptibility to fungal biotrophs by generating a green island around infection zones [102,103]. Although CKs are undoubtedly involved in defence mechanisms against biotic stress, their production often accompanies attack by particular phytopathogens. There is evidence that the reduction in photosynthesis in infected leaves results from increased invertase activity [103]. Paradoxically, infection seems to have a positive effect on antisenescent processes in the host plant as part of the protection of chlorophyll against disintegration (green islands). This antisenescent “improvement” is probably induced by the pathogen to gain more plant material for spreading its infection. However, although we know some of the ways by which these pathogens affect antisenescent responses, much remains unknown. 

## 7. CKs and Photosynthesis

The main function of leaves is to provide assimilates for plant growth through photosynthesis. CKs affect the functional as well as the structural aspects of photosynthesis at several levels. CKs induce cell division and differentiation even in the early stages of leaf development. Chernyaev et al. studied and reviewed the effect of CKs at the level of the whole leaf and found that they changed the leaf structure to have a greater number of cells per leaf area [104] and a larger number of vascular bundles and xylem and phloem elements [105]. Another structure closely connected to photosynthesis at the leaf level is the stomata. CKs acting as antagonists of abscisic acid can increase stomatal conductance [61,92,106,107,108] and, thus, modulate leaf gas exchange and the availability of CO_2_—the essential substrate for photosynthetically active tissue [6]. 

At the cellular level, CKs have a major effect on chloroplasts. As early as 1969, Boasson and Laetsch [109] reported that when etiolated tobacco leaves were transferred to light, the application of CKs increased the number of chloroplasts. Further research confirmed that CKs promoted the differentiation of etioplasts, their transition to chloroplasts [110,111,112], chloroplast division [113] and, finally, increased the number of chloroplasts [114]. In the presence of BAP, greening and plastid biogenesis in *Lupines lutus* and *Cucumis sativus* were substantially promoted [115,116]. Concurrently, a higher concentration and slower degradation of light-sensitive protochlorophyllide oxidoreductase, a key enzyme in chlorophyll biosynthesis, was observed in *Lupines lutus* and *Cucumis sativus* cotyledons [115,116]. 

At the level of the thylakoid membrane, it has been observed that CKs promote grana formation [110,117,118] and increase the content of photosynthetically active pigments [118,119,120] and starch grains [117,121,122,123,124]. CK regulation of chloroplast development, chlorophyll biosynthesis and nuclear plastid-related as well as plastid genes have been reviewed recently (for further reading, see [125]). CKs are reported to affect pigment-protein complexes involved in the light (primary) phase of photosynthesis, as well as enzymes of the dark (secondary) phase. Of the genes most upregulated by CKs, the most widely documented genes are those coding for the light-harvesting chlorophyll *a/b* binding proteins of photosystem II (*CAB*) and small and large subunits of RUBISCO (*RBCS*, *RBCL*) [112,126,127].

A close relationship between CKs and chloroplasts was confirmed by work showing that chloroplasts contain CKs of endogenous origin [128,129,130]. Chloroplasts of *Arabidopsis* contain four of seven IPT enzymes (AtIPT1, AtIPT3, AtIPT5 and AtIPT8) that catalyse the rate-limiting step of CK biosynthesis [129]. Senescence as the final stage in leaf development is accompanied by a decrease in CK content. Ananieva et al. [131,132] showed that progression of senescence of zucchini cotyledons correlated with a gradual decrease in the concentration of physiologically active CKs, together with an increase in the storage CK O-glucosides. Similarly, the content of active CK forms decreased in detached wheat and *Arabidopsis* leaves senescing in darkness [133,134].

As the photosynthetic apparatus is a possible source of ROS that may lead to destruction of assimilates that are intended for transport to growing or store tissue, its decomposition during senescence is highly regulated. In this regulation, CKs again play an important part. During senescence, changes in the chloroplast ultrastructure are well-documented, such as alterations in size and shape, disorganization of the thylakoid membrane, increased number of plastoglobuli and decreased chlorophyll content and photosynthesis function (for a review, see [135]). Application of exogenous CKs usually slows down typical senescence-induced changes, e.g., chlorophyll content decrease [119,123,136,137,138], plastoglobuli formation [118,121,139] and drop in key photosynthetic parameters, such as the rate of CO_2_ assimilation (*A*) [6,87,92,140,141], photochemical quenching (qP) and maximal photochemical efficiency of PSII (*F*_v_/*F*_m_) [87,91,92,123,142,143]. The senescence decelerating effect of CKs is not only connected to the upregulation of genes for biosynthesis and protection, however also to the downregulation of *SAGs* [144,145] and CK-mediated inhibition of degrading enzymes (e.g., activity of chlorophyllase, Mg-dechelatase, chlorophyll degrading peroxidase [146], RNase activity [147] and expression of pheophytinase [148]).

The effect of each CK ligand depends not only on its structure and concentration, however it also may be species-specific. Various CKs show modified ligand affinity for their receptors, e.g., in *A. thaliana*, *Brasica napus*, maize and potato [22,149,150,151]. The diverse effects of particular CKs regarding physiological responses to exogenous application have also been documented. Whereas BAP is widely known for its antisenescence effect on leaves of, e.g., broccoli, bean, barley, maize and wheat [85,87,137,152,153], after application to lettuce, no or only a weak effect was found [152,154]. Similarly, kin has repeatedly been reported as very active in senescence deceleration (e.g., [155,156,157]), however it showed an ambiguous effect on *Anthurium* [82]. It should be noted that the CK effect also depends on the age of cells and leaves [158] and light conditions [123,158,159].

## 8. Mechanisms and Genes Implicated in the Antisenescent and Antioxidant Activity of CKs

The ability of plants to perceive CKs acts through a modified bacterial two-component pathway that functions via a multi-step phosphorelay [160]. CK molecules in *Arabidopsis* are perceived by sensor histidine kinase receptors, named AHK2, AHK3 and CRE1/AHK4 [161,162,163]. Gene responses to CK treatment have been examined and reported a number of times [93,161,164]. The first genes to be induced by CKs (IBC genes), now known as A response regulator genes ARR5 and ARR4, were identified in 1998 [161,165]. AHK3 in particular was found to be the major *Arabidopsis* CK receptor connected with the antisenescent effect of CKs. It also plays a major role in CK-mediated leaf longevity through specific phosphorylation of a type-B response regulator, ARR2 [23,166]. Zwack et al. [167] showed that cytokinin response factor 6 (CRF6), the expression of which is downstream of the perception of CKs by AHK3, is involved in negative regulation of senescence. Recent research demonstrated that AHK2 and AHK3 are implicated in the routine repair of D1 protein, which is necessary for the functioning of photosystem II (PSII). This protective function of CKs during light stress depends on type B ARR1 and ARR12 [168]. On the other hand, the receptor CRE1/AHK4 probably affects oxidative damage during senescence. Janečková et al. [134] studied *Arabidopsis* mutants with two non-functional and only one functional CK AHK receptor and observed that the dark senescence induced increase in lipid peroxidation was retarded in the mutant with solely functional CRE1/AHK4 and also partially in the mutant with solely functional AHK2 receptor. Different patterns of hormonal regulation suggest that CKs may act transcriptionally to alter responses to ROS that are often produced during abiotic stress [93]. Over the last 10 years, using the model *Arabidopsis* plant, several transcriptomic datasets related to CKs have been generated from different technological platforms, culminating in RNA sequencing [164]. It is well-known today that both exogenous application of active CKs and an increase in their endogenous content can delay senescence [6,134,169]. Current molecular genetics strategies to manipulate leaf senescence are based either on enhancing CK production [6,7] and perception [170] or exogenous application of CKs and their derivatives [6,171]. It was shown that it is possible to genetically engineer IPT plants to overproduce CKs [6,7]. These IPT plants as well as exogenously added CKs, e.g., BAP, can be used in vegetables such as broccoli to effectively delay senescence as well as postharvest senescence owing to their antagonistic effect on ethylene [172]. Functional analysis of differentially expressed genes in BAP supplied postharvest broccoli showed regulation in the expression of genes involved in CK signalling, nutrient transport and photosynthesis. The addition of BAP caused downregulation of the gene responsible for ethylene synthesis [172]. Flowers of *Petunia x hybrida* transformed with PSAG12-IPT that overproduced endogenous CKs during the senescence period and produced the same amount of ethylene as wild-type flowers, however were less sensitive to exogenous ethylene and had a much longer lifetime [173]. This demonstrates that endogenous CKs lower the sensitivity of plants to ethylene and extend their lifetime. 

Many genes associated with senescence (SAGs) have been identified [174]. SAGs are genes that are expressed during senescence [170] that encode for degradative enzymes, such as RNAses [175], proteinases [176,177,178] and lipases [179], and products involved in nutrient translocation processes [180]. Temporal control of senescence was achieved when the promoter of the SAG12 gene was linked to an IPT gene [94]. The SAG12::IPT autoregulatory senescence inhibition system has also been successfully implemented in a number of important crops, such as rice, ryegrass, tomato, alfalfa, cauliflower, wheat, cassava and cotton [171,181]. Currently, it is used to delay senescence in green vegetables, such as lettuce and broccoli [94]. P_SAG12_-IPT tobacco plants that were grown in a growth-limiting nitrogen supply showed delayed senescence-associated declines in nitrogen, protein and Rubisco levels and photosynthesis rates [182]. Weaver and Gan published the expression of several *Arabidopsis thaliana* SAGs in attached and/or detached leaves and compared the response to age, dehydration, darkness, abscisic acid, cytokinin (BAP) and ethylene treatment [144]. BAP inhibited SAG expression in dark detachment experiments and the inhibition was generally greater in younger leaves. Thus, there is an indisputable connection between CK action and the light influence on senescence as the key factors in senescence regulation [134]. Lara et al. demonstrated that CKs upregulate the expression of extracellular cell wall invertase and that this enzyme is required for the delay of senescence caused by CKs [183]. This enzyme plays a crucial role in source-sink regulation, which has been shown to be a key molecular mechanism in CK senescence inhibition [183]. Vylíčilová et al. prepared a series of 2,6-disubstituted ARCK derivatives in 2016 and investigated the relation between the antisenescent effect of these compounds derived from CKs and their influence on photosystem II [44]. For most of the active compounds, regulation of gene expression in senescent *Arabidopsis* leaves was observed. In agreement with previously published data, it was shown that CK derivative treatment upregulated CK response regulators and other CK responsive genes, such as ARR15, ARR5, ARR8, ARR7, ARR4, ARR6, ARR9, CKX4, CRE1/AHK4, CRF2 and CRF5, however also genes encoding components of the photosystem II light harvesting complex (LHCII), i.e., At2g05070, At5g54270, At1g44575, At3g55330 and At2g39470, even though BAP had a negligible effect on these genes [44]. This supports the abovementioned suggestion of the need for electronegative atoms (which BAP lacks) in the vicinity of N6H and N7H. 

Transgenic plants have also been used to examine the influence of endogenous CKs on antioxidant defence systems. CKs induced the activity of antioxidant enzymes in transgenic plants, e.g., during plant ontogeny of P*ssu*-ipt tobacco [184]. Whereas the control plants showed a decline in total CK content, the transgenic plants exhibited at least a 10 times higher content of CKs than the controls, especially of Z and ZR. The transgenic plants also showed elevated activity of antioxidant enzymes, such as glutathione reductase (GR), SOD and APX [184]. Old tobacco leaves of P_SAG12_-IPT plants had a much longer lifespan and the concentrations of antioxidants ascorbate and GSH were higher than in wild-type leaves. At the same time, the chlorophyll and protein contents together with photosynthetic rate were increased. The decline in activity of antioxidative enzymes APX, GR and SOD during senescence was slowed down by CKs that were produced in P_SAG12_-IPT plants [185]. It was shown that old leaves of P_SAG12_-IPT plants and their chloroplasts maintained higher physiological parameters than in the control due to the extension of the period of greater antioxidant protection [186]. In a field study, stay-green cv. P3845 of *Zea mays* with enhanced levels of endogenous CKs showed higher CAT and SOD activities than earlier senescent cv. Hokku 55 [187]. 

The effects of exogenous CKs on photosynthetic capacity and antioxidant enzyme activities were evaluated in WN6 (a stay-green wheat cultivar) and JM 20 (control wheat, [85]). WN6 reached a higher grain mass, mainly due to a higher photosynthetic rate resulting from a maximal quantum yield of PSII photochemistry. Exogenously applied BAP enhanced antioxidant enzyme activities and decreased MDA content, as well as increased endogenous *t*Z levels [85].

## 9. Conclusions

Here, we review a family of important plant growth regulators—CKs—that are inseparably linked with plant senescence. The emphasis is on the interconnections between the CK influence on the photosynthetic apparatus, determinable antisenescent properties measured by several bioassays, antioxidative enzyme regulation, levels of antioxidant secondary metabolites in a number of plants or their explants and genes involved in plant senescence and its regulation. We highlight several molecular aspects that may represent new connections in the mechanism of action of these amazing small molecules that are indisputably involved in plant defence against biotic and abiotic stress. The role of systematic study and synthesis of new CK derivatives in relation to their biological functions is discussed, especially their antisenescent properties, together with their structural changes in comparison to the original molecules. Links between changes in structure and biological activities, especially effects on photosynthetic apparatus, secondary metabolite production and senescence related gene involvement, are described with regard to the overall functions of CKs within plants. Common structural motifs in CK molecules that could serve as a guide to specifically why these molecules have antisenescent properties are outlined. The involvement and ability of biotic stresses to enter the transcriptional process of CK production are also mentioned. This is especially relevant to green island formation and the influence on photosynthesis, which continue to be important issues. However, many questions remain which need further exploration of these interesting molecules with ancient perception and regulatory systems.

## Figures and Tables

**Table 1:** Effects of cytokinins on levels of antioxidant related enzymes, secondary metabolites and antioxidant activity in different assays. Arrows show ↑ increase or ↓ decrease in concentration or activity.

Cytokinin	Plant	Concentrations of Antioxidant Related Enzymes, Secondary Metabolites and Antioxidant Activity	Reference
BAP	St John’s-wort (*Hypericum hirsutum* sc.)	↑hyperforin	[79]
	St John’s-wort (*Hypericum macalatum* sc.)	↑pseudohypericin, hyperforin	[79]
	Wheat (*Triticum aestivum* L.) leaves	↑CAT, ↑APX, ↓level of H_2_O_2_	[35]
	Litchi (*Litchi chinensis Sonn*). fruit	↑SOD, ↑CAT, ↑APX, DPPH assay ↓level of H_2_O_2_, ↓lipid peroxidation,	[83]
	Skullcap (*Scutellaria altisima*) explants	↑baicalin, ↑wogonoside ↓lipid peroxidation	[76]
	Eggplant (*Solanum melongena* L.) plants	↓lipid peroxidation, ↑SOD, ↑CAT, ↑POD, ↑APX	[84]
	Wheat (JM20) plants	↓lipid peroxidation, ↑SOD, ↑CAT, ↑POD, ↑APX	[85]
	Summer maize (hybrids DengHai605, Zheng-Dan958) plants	↓lipid peroxidation, ↑SOD, ↑CAT, ↑POD	[87]
	Rice (*Oriza sativa* cv. Taichung Native 1) leaves	↓lipid peroxidation	[88]
	Maerwilla (*Merwilla plumbea*) explants	↑phenolic acids (PA, VA)	[70]
iP		↑phenolic acid (CafA)	
*m*TR		↑phenolic acids (PA, FA, 4CA), ORAC	
*m*T		ORAC	
	*Banana* (*Musa* spp. AAA cultivar ‘Williams’) explants	↑total phenolics, ↑proanthocyanidins	[69]
*m*MTTHP		↑total phenolics, ↑total flavonoids, ↑proanthocynidins	
	Maerwilla (*Merwilla plumbea*) explants	↑phenolic acids (4CA, FA)	[70]
*t*ZR	Creeping Bentgrass (*Agrostis palustris* L.) plants	↓lipid peroxidation, ↓electrolyte leakage, ↑SOD, ↑CAT	[86]
	Creeping Bentgrass (*Agrostis palustris* L.) plants	↓lipid peroxidation, ↑SOD, ↑CAT	[89]
TDZ	Skullcap (*Scutellaria alpina*) explants	↑flavonoids (BC, WO) ↑verbascoside	[75]
CPPU	Maize (*Zea mays* L.) seedlings	↓lipid peroxidation, ↓level of H_2_O_2_, ↑CAT	[90]
	Tomato (*Lycopersicon esculentum* Mill.) leaves	↓lipid peroxidation, ↑SOD, ↑APX	[91]
INCYDE	Lettuce (*Lactuca sativa*)	↑4CA, ↑FA, ORAC	[78]
	*Eucomis autumnalis* explants	↑flavonoids, DPPH and β-carotene acid antioxidant assay	[77]
PI-55			
Kin	St John’s-wort (*Hypericum hirsutum* sc.)	↑hyperforin	[79]
	St John’s-wort (*Hypericum macalatum* sc.)	↑pseudohypericin, hyperforin	[79]
	Tomato (*Solanum lycopersicum* L.) plants	↓level of H_2_O_2_, ↓lipid peroxidation, ↓electrolyte leakage, ↑SOD, ↑CAT, ↑ascorbate-glutathione cycle, ↑total phenols, ↑flavonoids	[92]
	Oat (*Avena sativa* L. cv. Victory) leaves	↓lipid peroxidation, ↑SOD, ↑CAT,	[29]
	Anthurium (*Anthurium andraeanum* Lindl.) leaves	↑APX	[82]

4CA, 4-coumaric acid; APX, ascorbate peroxidase; BAP, 6-(benzylamino)purine; BC, baicalin; CAT, catalase; CafA, caffeic acid; CPPU, *N*-(2-chloropyridin-4-yl)-*N*′-phenylurea; DPPH, 2,2-diphenyl-1-picrylhydrazyl; FA, ferulic acid; INCYDE, 2-chloro-6-(3-methoxyphenylamino)purine; iP, 6-(2-isopentenylamino)purine; Kin, kinetin; mMTTHP, 6-(3-hydroxybenzylamino)purine-9-THP; mT, meta-topolin, 6-(3-hydroxybenzylamino)purine; mTR, 6-(3-hydroxybenzylamino)purine riboside; ORAC, oxygen radical absorbance capacity; PA, protocatechuic acid; PI-55, 6-(2-hydroxy-3-methylbenzylamino)purine; POD, peroxidase; sc, shoot culture; SOD, superoxide dismutase; TDZ, thidiazuron; *t*ZR, *trans*-zeatin riboside; VA, vanillic acid; WO, wogonoside.

## Abbreviations

2Cl-3F-BAPR	2-chloro-6-(3-fluorobenzylamino)-9-β-d-ribofuranosylpurine
3F-BAP	6-(3-fluorobenzylamino)purine
3F-BAPR	6-(3-fluorobenzylamino)-9-β-d-ribofuranosylpurine
AHK	*Arabidopsis* histidine kinase
APX	ascorbate peroxidase
ARCK	aromatic cytokinins
ARR	*Arabidopsis* response regulator
BAP	6-(benzylamino)purine
CAT	catalase
CK	cytokinin
CPPU	*N*-(2-chloropyridin-4-yl)-*N*′-phenylurea
CRF	cytokinin response factor
*c*Z	6-(*Z*)-(4-hydroxy-3-methylbut-2-enylamino)purine (*cis*-zeatin)
DCPPU	*N*-(2,6-dichloro-pyridin-4-yl)-*N*′-phenylurea
DHZ	6-(4-hydroxy-3-methylbutylamino)purine (dihydrozeatin)
DHZR	6-(4-hydroxy-3-methylbutylamino)-9-β-d-ribofuranosylpurine (dihydrozeatin-riboside)
GSH	glutathione
INCYDE	2-chloro-6-(3-methoxyphenylamino)purine
iP	6-(2-isopentenylamino)purine
IPT	isopentenyl transferase
ISCK	isoprenoid cytokinins
Kin	6-(furfurylamino)purine (kinetin)
MDA	malondialdehyde
*m*T	6-(3-hydroxybenzylamino)purine (meta-topolin)
*m*TR	6-(3-hydroxybenzylamino)-9-β-d-ribofuranosylpurine (meta-topolin-9-riboside)
ORAC	oxygen radical absorbance capacity
*o*T	6-(2-hydroxybenzylamino)purine (ortho-topolin)
PI-55	6-(2-hydroxy-3-methylbenzylamino)purine
POD	peroxidase
ROS	reactive oxygen species
SAGs	senescence-associated genes
SOD	superoxide dismutase
TDZ	1-phenyl-3-(1,2,3-thiadiazol-5-yl)urea (thidiazuron)
THF	tetrahydrofuran-2-yl
THP	tetrahydropyran-2-yl
*t*Z	6-(*E*)-(4-hydroxy-3-methylbut-2-enylamino)purine (*trans*-zeatin)
*t*ZR	6-(*E*)-(4-hydroxy-3-methylbut-2-enylamino)-9-β-d-ribofuranosylpurine (*trans*-zeatin-9-riboside)
WLSA	wheat leaf senescence assay
Z	6-(4-hydroxy-3-methylbut-2-enylamino)purine (zeatin)
ZR	6-(4-hydroxy-3-methylbut-2-enylamino)-9-β-d-ribofuranosylpurine (zeatin-9-riboside)
